# Apilarnil Improves Early Histological Repair and Biomechanical Strength During Experimental Femoral Fracture Healing in Rats

**DOI:** 10.3390/bioengineering13080887

**Published:** 2026-07-31

**Authors:** Raşit Emin Dalaslan, Mehmet Arıcan, Meral Kekecoglu, Yalçın Turhan, Tuğçe Çaprazlı, Mehmet Gamsızkan, Zekeriya Okan Karaduman, Mücahid Osman Yücel, Sönmez Sağlam

**Affiliations:** 1 Orthopedics and Traumatology Department, Düzce University Medical Faculty, Düzce 81620, Turkey; ari_can_mehmet@hotmail.com (M.A.); karadumano@hotmail.com (Z.O.K.); mucahidyucel@duzce.edu.tr (M.O.Y.); dr.sonmezsaglam@gmail.com (S.S.); 2 Beekeeping Research Development and Application Centre, Department of Biology, Faculty of Science, Düzce University, Düzce 81620, Turkey; meralkekecoglu@duzce.edu.tr; 3 Orthopedics and Traumatology Department, Health Sciences University, Bilkent City Hospital, Ankara 06800, Turkey; yturhan_2000@yahoo.com; 4 Beekeeping Program, Düzce Vocational School, Düzce University, Düzce 81620, Turkey; tcaprazli@gmail.com; 5 Department of Pathology, School of Medicine, Düzce University, Düzce 81060, Turkey; drgamsiz@yandex.com.tr

**Keywords:** apilarnil, biomechanics, fracture healing, histopathology, bone regeneration

## Abstract

Fracture healing remains a major challenge in orthopedic practice, and natural bioactive products have attracted increasing interest as supportive agents for bone regeneration. Apilarnil (drone larvae homogenate), a bee-derived natural product with antioxidant and anti-inflammatory properties, has not previously been investigated in fracture healing. This study evaluated the effects of oral apilarnil on experimental femoral fracture healing in rats. Forty-two male Wistar Albino rats underwent standardized closed femoral fracture fixation with intramedullary Kirschner wire stabilization and were randomly assigned to either a control group or an apilarnil-treated group receiving 400 mg/kg/day orally. Animals were sacrificed on postoperative days 15, 30, and 45. Fracture healing was assessed radiologically using the Lane and Sandhu scoring system, histopathologically using the Huo scoring system, and biomechanically by a three-point bending test. Histopathological scores were significantly higher in the apilarnil group on postoperative day 15 (*p* = 0.042), whereas no significant differences were observed at later time points. Biomechanical testing demonstrated significantly greater fracture strength in the apilarnil-treated group on postoperative days 30 (*p* = 0.001) and 45 (*p* = 0.004), while radiological scores did not differ significantly between groups throughout the study. These findings suggest that apilarnil may support fracture healing by enhancing early tissue repair and improving the biomechanical properties of healing bone, warranting further experimental and clinical investigation.

## 1. Introduction

One of the most interesting topics in Orthopedics and Traumatology is fracture healing and consolidation, an area in which a large number of both in vitro and in vivo studies have been conducted [1,2]. Especially in elderly individuals, bone healing is slow due to complications such as low bone quality, osteoporosis, reduced bone formation, delayed periosteal reaction and cellular differentiation, impaired osteogenesis, as well as a decrease in the number and proliferative capacity of mesenchymal stem cells. These factors significantly reduce patients’ quality of life [3,4]. Hormonal factors such as estrogen and growth hormone, vitamins D and C, and essential amino acids play an important role in the fracture healing process [5]. In elderly patients and in younger patients with impaired bone regeneration, nutritional or pharmacological supplements that enhance bone metabolism are frequently used to stimulate and accelerate fracture healing [6].

Bone fractures occur with increasing frequency in both young and elderly populations due to traffic and occupational accidents, falls from height, and metabolic bone diseases such as osteoporosis. These fractures may result in loss of employment, permanent disability, and high treatment costs [7,8]. In recent years, emphasis has been placed on the development of natural and domestic pharmaceutical products in order to reduce costs in the healthcare sector. In parallel with the growing interest in natural products and traditional and complementary medicine, research on the potential use of natural substances to promote fracture healing or strengthen bone structure has gained increasing attention [9,10].

The medical importance of honeybee products among natural substances has been demonstrated by numerous scientific studies, particularly since the late twentieth century, and they have become widely used for both preventive and therapeutic purposes [11,12]. Honeybee products such as propolis, royal jelly, honey, bee venom, pollen, and apilarnil, which has become a popular dietary product in recent years, have long been used in traditional medicine for the treatment of various diseases. Apilarnil, also referred to as drone larvae homogenate (DLH) or drone brood homogenate, is a bee-derived product obtained from drone larvae. Scientific studies have shown that these products possess biological activities including antimicrobial, antioxidant, antitumor, anti-inflammatory, and antiulcer effects [13,14,15]. Drone larvae are consumed as a nutritious food in many cultures and are a rich source of protein, essential amino acids, vitamins A, B, C, and D, as well as minerals such as calcium, magnesium, iron, potassium, and phosphorus [12,13].

A review of the literature reveals that only a limited number of experimental studies have investigated the medical use of apilarnil. To the best of our knowledge, no study has yet evaluated its effects on the fracture healing process. Therefore, this study represents the first experimental investigation of the effects of apilarnil on fracture healing. The aim of this experimental study was to evaluate the radiological, histopathological, and biomechanical effects of apilarnil on fracture healing. However, it should be noted that this study was conducted using young, healthy rats, which may limit the generalizability of the findings to osteoporotic or elderly populations.

## 2. Materials and Methods

In this study, 42 male Wistar Albino rats with a mean age of 2.5 months (range, 2–3 months) and a mean body weight of 250 g (range, 200–300 g) were used. An a priori sample size calculation was performed using G*Power software (version 3.1; Heinrich Heine University, Düsseldorf, Germany). Based on the expected large treatment effect in this exploratory experimental study, a large effect size (Cohen’s f = 0.60), a significance level (α) of 0.05, a statistical power of 80% (1 − β = 0.80), and six experimental groups were assumed. The minimum required sample size was calculated as 42 animals (7 animals per subgroup). Prior to the study, ethical approval was obtained from the Düzce University Faculty of Medicine Experimental Animals Local Ethics Committee (Approval No. 2019/1/5; approval date: 19 February 2019). The study was conducted at the Düzce University Faculty of Medicine Experimental Animal Application and Research Center. All experimental procedures were performed in accordance with institutional guidelines, the ARRIVE guidelines, and the Principles of Laboratory Animal Care (NIH Publication No. 85-23, revised 1996). Animal care and experimental procedures complied with national and institutional regulations for the protection and use of laboratory animals.

The animals were randomly divided into two groups, with seven rats housed per cage, and were acclimatized to the laboratory environment for one week before surgery. Throughout the study, the rats had free access to tap water and were fed standard laboratory rodent chow ad libitum. The animals were housed in a controlled environment with a temperature of 23–25 °C and a 12:12 h light-dark cycle. No antibiotic prophylaxis was administered to any group. No postoperative mortality or wound infection was observed in any of the animals.

Apilarnil (drone larvae homogenate [DLH]) was obtained from *Apis mellifera* drone larvae. Readymade honeycombs containing drone cells were placed in beehives during the early spring period when egg laying was high. The combs were removed after 7–8 days, and 3–5-day-old drone larvae were collected into sterile containers using a vacuum larva collection device. The samples were immediately transported to the laboratory under cold chain conditions. In the laboratory, the collected larvae were placed in sterile Petri dishes, frozen, and stored at −20 °C. Lyophilization was performed at −50 °C under reduced pressure for 2–3 h. The lyophilized material was then ground into powder using a sterile mortar and stored at +4 °C in daily doses until administration [14]. To minimize batch-to-batch variability, all apilarnil used throughout the experiment was prepared from a single production batch, lyophilized under identical conditions, aliquoted into daily doses, and stored under the same storage conditions until administration. Apilarnil is known to contain various bioactive compounds, including proteins, fatty acids, vitamins, and phenolic components, as reported in previous studies [16,17]. However, no specific biochemical characterization of the homogenate used in this study was performed, as it was intentionally used as a whole natural product without further chemical fractionation in order to reflect its traditional and practical use.

Following the surgical procedure, the 42 rats were divided into two groups: a control group (n = 21) and an apilarnil-treated group (n = 21). Each group was further subdivided into three subgroups, with seven rats per subgroup, scheduled for sacrifice on postoperative days 15, 30, and 45. The control group received routine postoperative care, whereas the treatment group was administered apilarnil by oral gavage at a dose of 400 mg/kg/day once daily throughout the study period [18].

After sacrifice, the right femurs were disarticulated from the hip and knee joints. Soft tissues were carefully removed without damaging the callus tissue. All femurs were subsequently evaluated using radiological, biomechanical, and histopathological methods. Radiological and histopathological evaluations were performed by observers blinded to the study groups.

### 2.1. Surgical Technique

After completion of the necessary preoperative preparation and monitoring, the rats were transferred to the operating room. The anesthetic dose was calculated individually by weighing each rat using an electronic scale. General anesthesia was induced with an intraperitoneal injection of ketamine (50 mg/kg; Eczacıbaşı, İstanbul, Turkey) and xylazine (10 mg/kg; Bayer, İstanbul, Turkey). The injection was administered through the left inguinal region. The right knee region was shaved and disinfected with povidone iodine solution (Batticon^®^, ADEKA, Istanbul, Turkey).

A 1 cm longitudinal anteromedial skin incision was made. The joint capsule was opened medially to the patella, and the knee was flexed by laterally displacing the patella. The intercondylar region of the distal femur was exposed, and a 1.2 mm Kirschner wire (Tıpmed^®^, İzmir, Turkey) was inserted between the femoral condyles and advanced proximally until it exited the proximal femur. The portion of the wire remaining within the medullary canal was cut flush with the femoral condyles to prevent protrusion. The knee was then extended, the patella was reduced to its anatomical position, and the joint capsule was closed with 3-0 Vicryl sutures. The skin was closed using 2-0 silk sutures.

**Figure 1:**
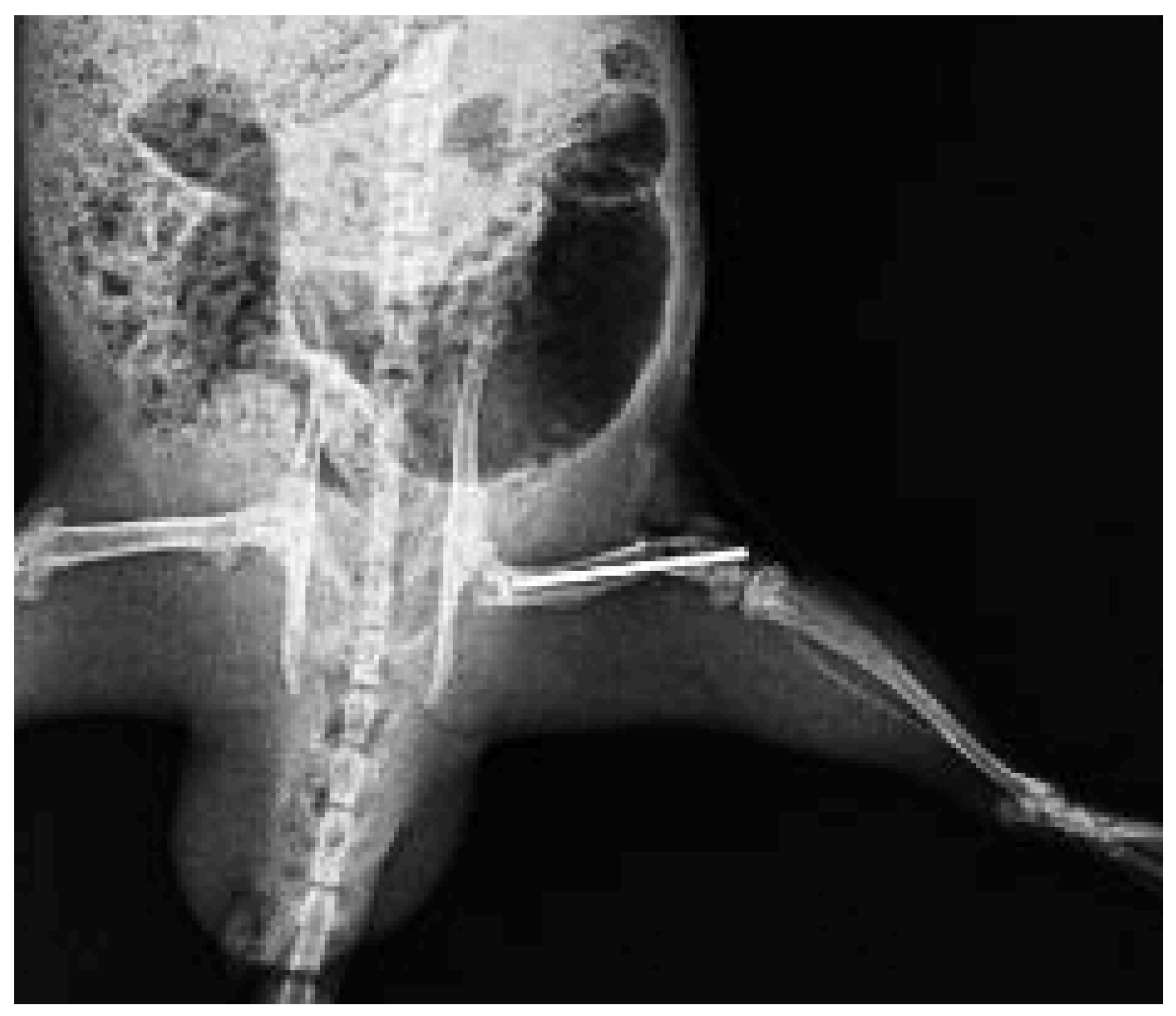
Representative anteroposterior radiograph obtained immediately after surgery demonstrating a standardized transverse fracture of the right femoral mid-diaphysis stabilized with an intramedullary Kirschner wire (K-wire). The radiograph was obtained to confirm successful fracture creation and appropriate intramedullary fixation.

Following wound closure, the surgical site was cleansed with povidone iodine. A standardized closed transverse fracture of the right femoral mid-diaphysis was then created using a guillotine-type fracture apparatus, as described by Bonnarens and Einhorn [19]. Postoperative analgesia was provided using fentanyl citrate (0.02 mg/kg, subcutaneously; Fentanest^®^, Türkem İlaç, Ankara, Turkey) for three consecutive days following surgery. All animals were closely monitored for signs of pain or distress throughout the postoperative period. Fracture formation was immediately confirmed by clinical examination and plain radiographic imaging (Figure 1). The intramedullary K-wire remained in place throughout the healing period to ensure fracture stabilization. It was carefully removed only prior to biomechanical testing to avoid interference with the three-point bending measurements.

### 2.2. Radiological Examination

**Figure 2:**
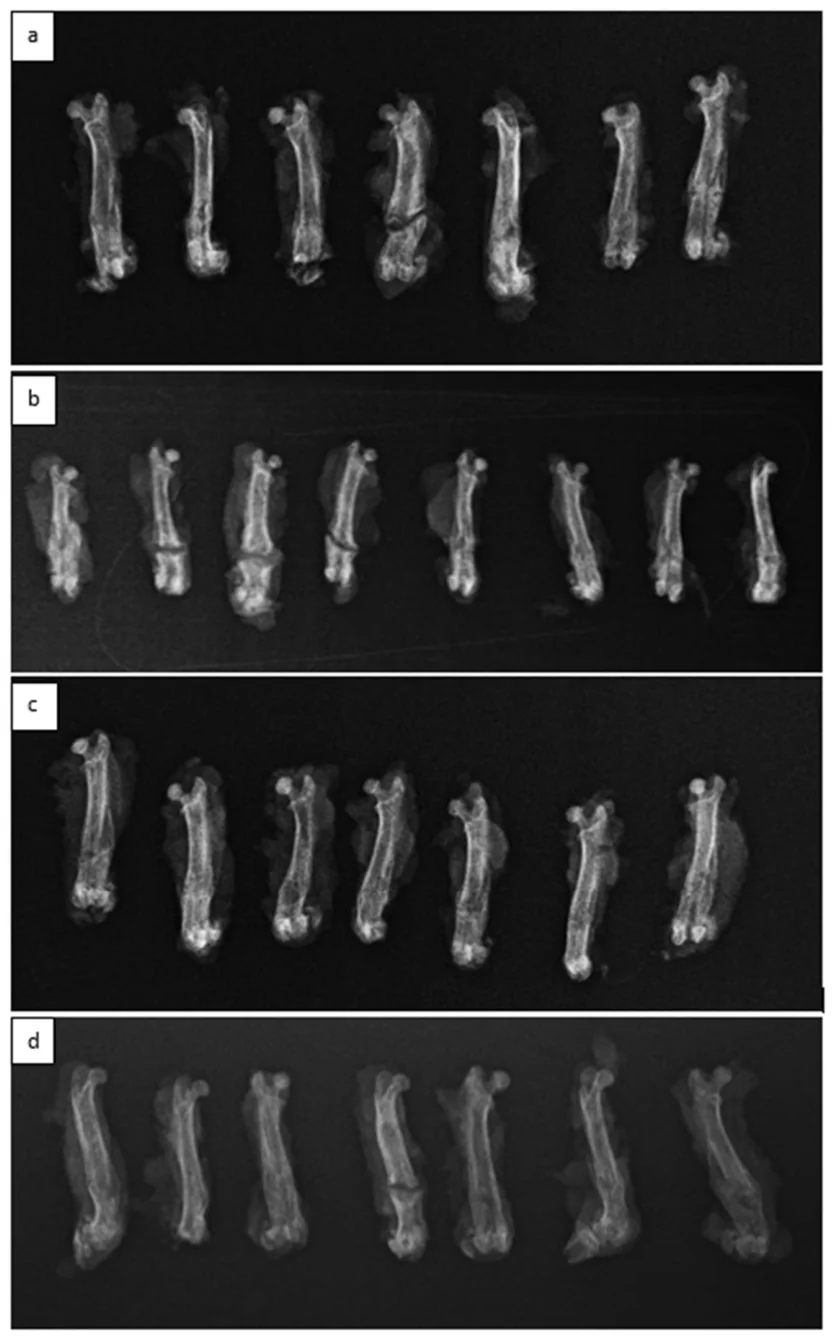
Representative radiographs of the control and apilarnil-treated groups during fracture healing. (**a**) Control group, day 30. (**b**) Apilarnil-treated group, day 30. (**c**) Control group, day 45. (**d**) Apilarnil-treated group, day 45.

Radiographic evaluations were performed on all femurs harvested from rats sacrificed on postoperative days 15, 30, and 45 (Figure 2). Radiological scoring was conducted using the Lane and Sandhu grading system [20]. According to this system, fracture healing was graded based on radiographic findings as follows: 0, no evidence of healing; 1, callus formation; 2, initiation of bony union; 3, disappearance of the fracture line; and 4, complete bony union. Radiographs were evaluated independently by two blinded orthopedic surgeons. In cases of disagreement, the final radiological score was determined by averaging the two observers’ scores. Interobserver agreement was assessed using weighted Cohen’s kappa.

### 2.3. Biomechanical Analysis

Biomechanical analysis was performed on 28 rat femurs harvested on postoperative days 30 and 45 from both the control and apilarnil-treated groups. Although biomechanical testing was initially planned for femurs harvested on postoperative day 15 in both groups, these samples (n = 14) were excluded from biomechanical analysis due to the presence of only soft callus formation. All biomechanical tests were performed on the day of sacrifice without delay. Prior to testing, the intramedullary fixation material was carefully removed.

Biomechanical testing was conducted using a BESMAK BMT E Series material testing device (Besmak, Ankara, Turkey) at the Düzce University Scientific and Technological Research Application and Research Center. A three-point bending test was applied for biomechanical evaluation. Before testing, the femurs were positioned horizontally on the testing device, and the distance between the two support points was standardized to 18 mm for all specimens, taking femoral length into consideration. Subsequently, a bending force was applied at a constant displacement rate of 3 mm/min, with the fracture line aligned at the midline between the support points. The applied force was gradually increased until structural failure occurred, and the maximum load to failure (N) recorded by the device was documented for each specimen.

### 2.4. Histopathological Examination

**Figure 3:**
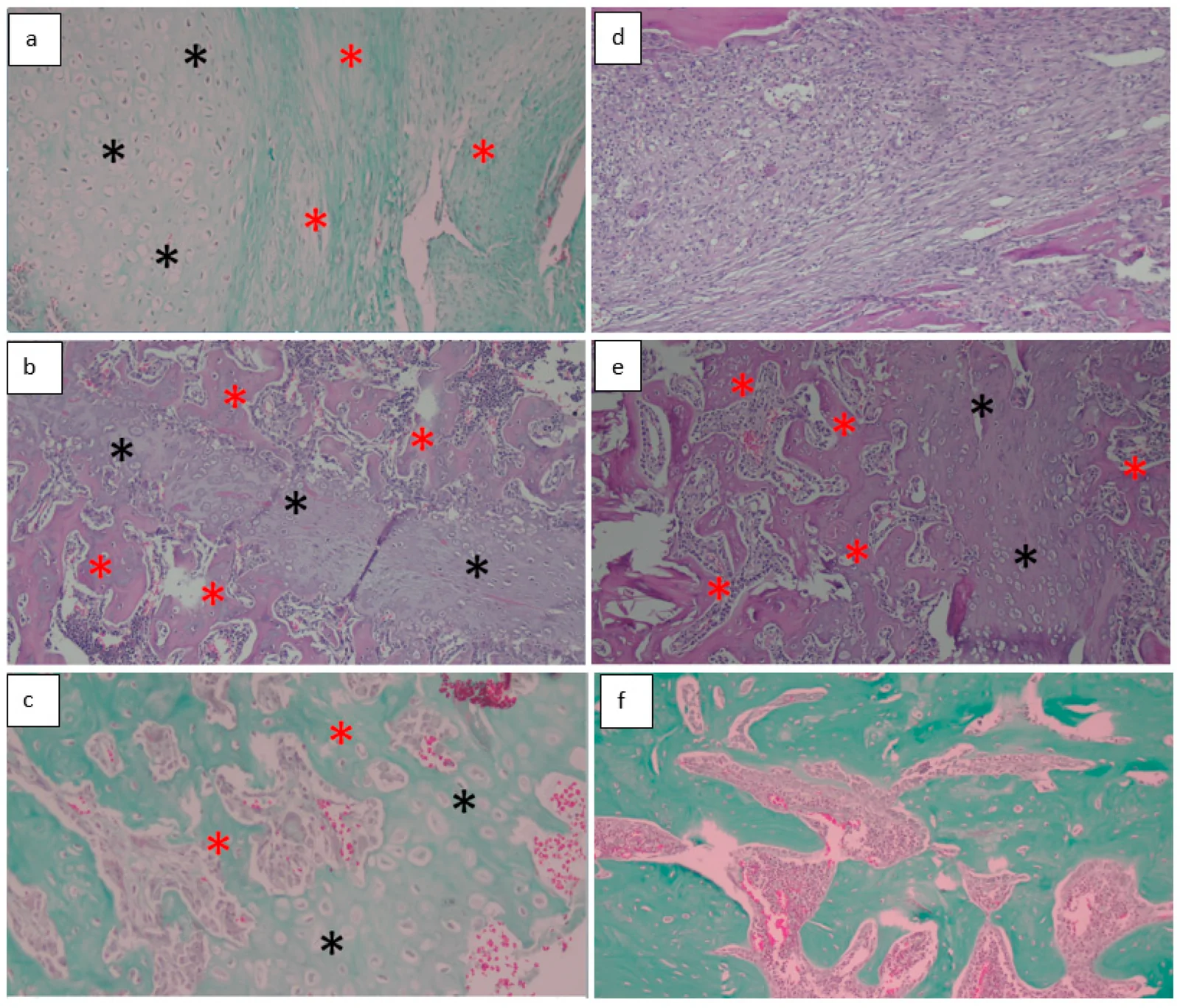
Representative histopathological sections of the control and apilarnil-treated groups during fracture healing. (**a**) Control group, day 15 (Score 3): equal amounts of cartilage (black stars) and fibrous tissue (red stars) (Masson’s trichrome, ×200). (**b**) Control group, day 30 (Score 7): equal amounts of cartilage (black stars) and immature bone (red stars) (H&E, ×100). (**c**) Control group, day 45 (Score 7): equal amounts of cartilage (black stars) and immature bone (red stars) (Masson’s trichrome, ×200). (**d**) Apilarnil-treated group, day 15 (Score 1): fibrotic connective tissue (H&E, ×100). (**e**) Apilarnil-treated group, day 30 (Score 8): predominantly immature bone (red stars) with a smaller amount of cartilage (black stars) (H&E, ×100). (**f**) Apilarnil-treated group, day 45 (Score 10): mature bone tissue (Masson’s trichrome, ×100).

The soft tissues surrounding all fractured femurs were carefully removed without damaging the periosteum. For histopathological evaluation, two sections were obtained from each specimen. The femoral specimens were fixed in 4% formaldehyde at 4 °C for 48 h and subsequently decalcified in 7% formic acid. Following decalcification, the samples were embedded in paraffin blocks, and 7 μm thick sections were obtained. The sections were stained with hematoxylin and eosin. Histopathological evaluation of fracture healing was performed by an experienced pathologist (MG) using the scoring system proposed by Huo et al., with all samples assessed in a blinded manner with respect to both the experimental groups and the time points [21] (Figure 3).

### 2.5. Statistical Analysis

In this study, statistical analyses were performed with the NCSS (Number Cruncher Statistical System) 2007 Statistical Software (Kaysville, UT, USA) package. In evaluating the data, the distribution of variables was examined with the Shapiro–Wilk normality test as well as descriptive statistical methods (mean, standard deviation, median, interquartile range). Friedman Test was used for time comparisons of variables that did not show normal distribution, Dunn’s multiple comparison test was used for subgroup comparisons, and Mann Whitney U test was used for comparison of independent groups. Interobserver agreement for radiological scoring was assessed using the quadratic weighted Cohen’s kappa coefficient. The results were evaluated at the significance level of *p* < 0.05.

## 3. Results

**Figure 4:**
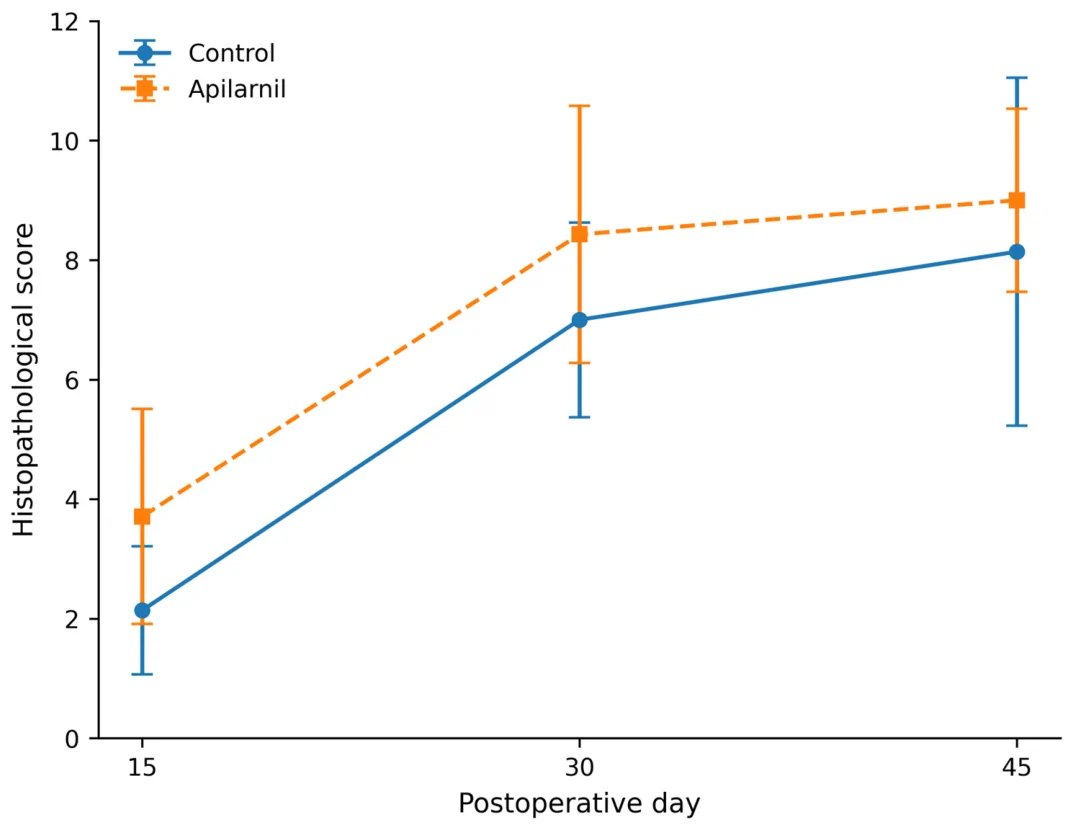
Histopathological comparison of the control and apilarnil-treated groups on postoperative days 15, 30 and 45.

Histopathological evaluation showed that the mean score on postoperative day 15 was significantly higher in the apilarnil group compared with the control group (control: 2.14 ± 1.07; treatment: 3.71 ± 1.80; *p* = 0.042). However, no statistically significant differences were observed between groups on postoperative days 30 and 45 (day 30: control: 7.00 ± 1.63; treatment: 8.43 ± 2.15; *p* = 0.079; day 45: control: 8.14 ± 2.91; treatment: 9.00 ± 1.53; *p* = 0.631) (Table 1, Figure 4).

**Figure 5:**
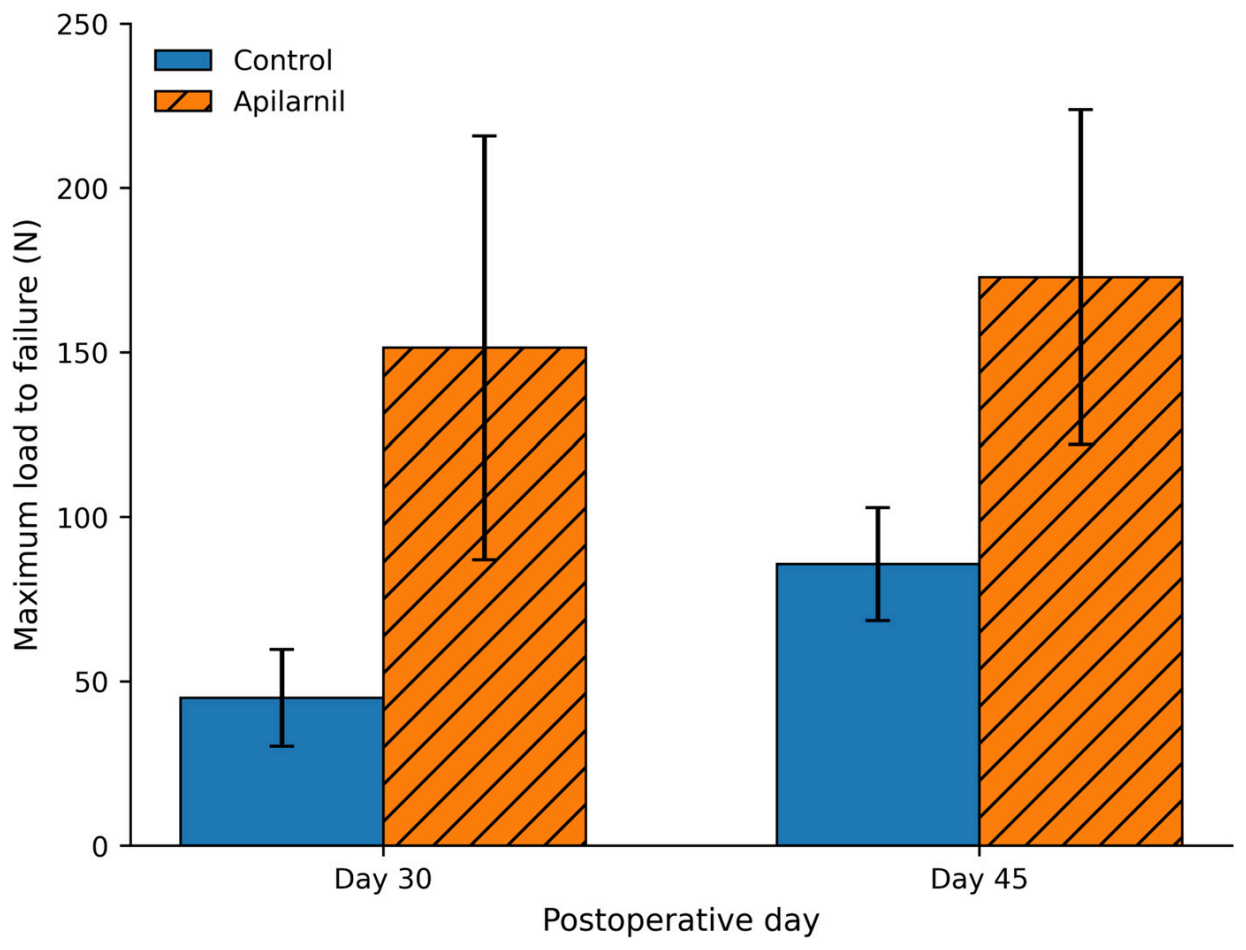
Biomechanical comparison of the control and apilarnil-treated groups on postoperative days 30 and 45.

Biomechanical evaluation demonstrated that the mean values on postoperative days 30 and 45 were significantly higher in the apilarnil group compared with the control group (day 30: control: 45.0 ± 14.7 N; treatment: 151.4 ± 64.4 N; *p* = 0.001; day 45: control: 85.7 ± 17.2 N; treatment: 172.9 ± 50.9 N; *p* = 0.004) (Table 2, Figure 5).

**Figure 6:**
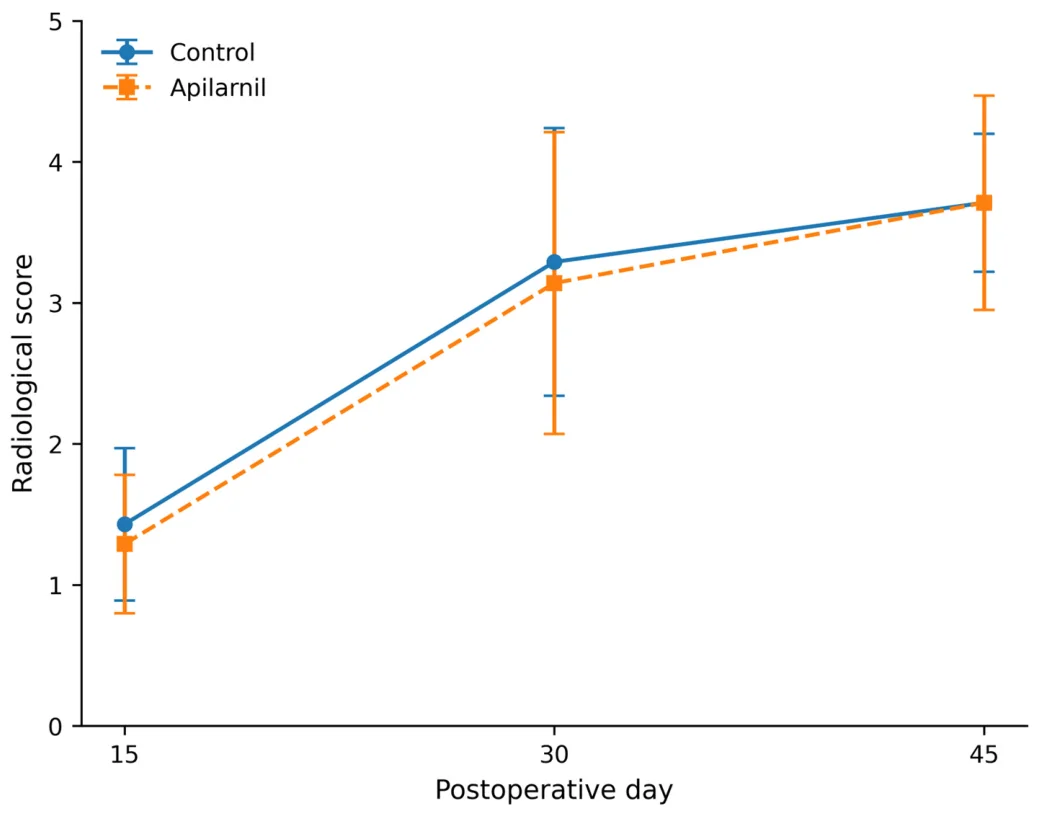
Radiological comparison of the control and apilarnil-treated groups on postoperative days 15, 30 and 45.

Radiological evaluation revealed no statistically significant differences between the control and apilarnil groups on postoperative days 15, 30, and 45 (day 15: control: 1.43 ± 0.54; treatment: 1.29 ± 0.49; *p* = 0.591; day 30: control: 3.29 ± 0.95; treatment: 3.14 ± 1.07; *p* = 0.782; day 45: control: 3.71 ± 0.49; treatment: 3.71 ± 0.76; *p* = 0.656) (Table 3, Figure 6). Interobserver agreement for radiological scoring was excellent (quadratic weighted Cohen’s κ = 0.885).

## 4. Discussion

Fracture healing is a complex biological process involving the coordinated interaction of multiple biological, mechanical, and environmental factors, and various treatment, physical, and biological therapy modalities influence different stages of this process [22,23]. Although the number of scientific studies on fracture healing has increased steadily, the underlying mechanisms are still not fully understood, and the factors that positively influence this process have not yet been completely elucidated [24,25,26,27].

Honeybee products, which constitute important natural components of the human diet, have long been used to manage various health conditions, particularly in regions such as Asia, Africa, and South America [12,13]. Although previous studies have investigated the effects of honey and propolis on bone healing, no research evaluating the contribution of drone larvae to the fracture healing process has been identified in the literature [10,28]. 

Apilarnil has recently gained considerable attention because of its complex chemical composition and rich nutritional profile. Previous phytochemical and compositional analyses have shown that apilarnil contains high concentrations of proteins, free amino acids, unsaturated fatty acids, vitamins (particularly vitamins A, B-complex, C, and D), minerals, carbohydrates, phospholipids, sterols, and a wide range of phenolic and flavonoid compounds [11,12,13,14,15,16,17]. Among its major lipid constituents, oleic acid and palmitic acid have been identified as the predominant fatty acids, whereas quinic acid, fumaric acid, kaempferol, and quercetin are among the principal bioactive phytochemicals reported in recent analytical studies [16,17]. Owing to this diverse biochemical composition, experimental studies have demonstrated that apilarnil exhibits a broad spectrum of biological activities, including antimicrobial, antioxidant, anti-inflammatory, nephroprotective, hepatoprotective, estrogenic, androgenic, and neuroprotective effects, as well as protective effects against testicular injury [11,14,18,29,30,31,32]. The combination of these bioactive constituents may provide a favorable biological environment for tissue regeneration by reducing oxidative stress, modulating inflammatory responses, and supporting cellular proliferation and extracellular matrix synthesis, although the specific components responsible for the observed effects in fracture healing remain to be elucidated.

In a comprehensive review by Korani et al. on honeybee-derived products in bone tissue engineering, derivatives such as honey, propolis, royal jelly, beeswax, and bee venom were reported to have potential benefits in supporting bone health [9]. In an experimental study by Güney et al. evaluating the effects of propolis on fracture healing, rats receiving oral propolis demonstrated higher bone mineral density, improved radiological and histological scores, and reduced oxidative stress markers, including superoxide dismutase, total glutathione, and myeloperoxidase, compared with untreated controls. The authors concluded that propolis exerted time-dependent beneficial effects on fracture healing [28]. Similarly, Şahin et al. investigated the effects of grayanotoxin-containing honey, normal honey, and propolis on fracture healing and reported that both grayanotoxin-containing honey and propolis accelerated the fracture healing process [10].

A review of the orthopedic and traumatology literature reveals only one experimental study investigating the use of apilarnil, which focused on wound healing rather than bone repair [33]. In that study, Andritoiu et al. evaluated the effects of ointments containing honey, apilarnil, propolis, and their combination on cutaneous wound healing. Macroscopic assessment showed no significant differences among the groups in terms of wound diameter, re-epithelialization, or wound contraction. Histopathological evaluation demonstrated similar findings across incision, excision, and thermal burn models, including comparable lymphocyte and fibroblast counts accompanied by marked dermal edema. The authors concluded that formulations based on honey, propolis, and apilarnil all exhibited wound healing and re-epithelialization effects [33]. Despite these findings, no previous study has investigated the role of apilarnil in fracture healing. Therefore, the present study was designed based on the hypothesis that apilarnil, as a popular dietary supplement with rich molecular content, may enhance fracture healing.

Successful fracture healing requires precise coordination among multiple cell types and molecular pathways. Disruption of these tightly regulated processes may result in delayed union or nonunion, and fracture-related complications remain relatively common in orthopedic practice [3,25]. Consequently, there is a need for novel treatment strategies aimed at accelerating fracture healing. Such strategies should include easily accessible and safe natural nutritional supplements with osteoanabolic potential, in addition to conventional conservative and surgical treatments. In the present experimental study, complete fracture union was achieved by the end of the follow-up period in both the control group and the apilarnil group.

In the present study, the significantly higher histopathological scores observed in the apilarnil group on postoperative day 15 suggest that apilarnil may contribute to the early phase of fracture healing. This finding indicates a potential accelerating effect on the initial stages of callus formation and tissue organization, which are critical for successful fracture repair. The absence of significant differences between the groups at later time points, namely postoperative days 30 and 45, may imply that the beneficial effects of apilarnil are more pronounced during the early inflammatory and reparative phases of fracture healing rather than during the remodeling phase. Given the rich molecular composition of apilarnil, including essential amino acids, fatty acids, vitamins, and bioactive compounds, its early osteogenic and supportive effects on cellular activity and matrix formation may account for the observed histological improvement at postoperative day 15. These effects may be mediated through several biological pathways. Amino acids present in apilarnil may support osteoblast proliferation and extracellular matrix synthesis, which are essential for early callus formation [34,35]. In addition, fatty acids and phenolic compounds may modulate inflammatory responses and oxidative stress, both of which play critical roles in the fracture healing cascade [36,37]. However, the exact molecular mechanisms underlying these effects remain to be elucidated.

Biomechanical testing showed a tendency toward greater mechanical strength in the apilarnil group on postoperative days 30 and 45, while no statistically significant differences were observed in radiological scores between the groups at postoperative days 15, 30, or 45. These findings may suggest that biomechanical assessment could reflect certain qualitative aspects of bone healing that are not always apparent on conventional radiographic evaluation, particularly during the later phases of the healing process. 

The apparent differences among the histological, biomechanical, and radiological findings should be interpreted with caution, as these assessment modalities evaluate different aspects of fracture healing and exhibit different sensitivities throughout the healing process. Histological examination is capable of detecting early cellular and tissue-level changes before substantial mineralization occurs, whereas radiological evaluation primarily reflects mineralized callus formation and may be less sensitive during the early stages of repair. Similarly, biomechanical testing assesses the functional integrity of the healing bone, which may improve independently of detectable radiographic changes. Therefore, the lack of complete concordance among these outcome measures likely reflects the temporal and biological complexity of fracture healing rather than contradictory results. 

This interpretation is supported by previous experimental studies evaluating the relationship between radiographic findings and the mechanical properties of healing bone. Panjabi et al. demonstrated that conventional radiographic assessment alone was insufficient to accurately predict the mechanical strength of healing fractures under experimental conditions [38]. Likewise, Watanabe et al. reported that although radiographic findings correlated reasonably well with biomechanical recovery during the early stages of fracture healing, conventional radiographs tended to underestimate mechanical competence during the later stages of repair [39]. These observations suggest that radiological and biomechanical assessments provide complementary rather than interchangeable information, and may explain why significant improvements in mechanical strength were observed in the present study despite the absence of statistically significant radiographic differences between the groups.

Recent studies have drawn increasing scientific attention to apilarnil (drone larvae homogenate) due to its unique chemical composition and biological properties [10,12,13,16,17,29,30,31,33]. The diverse biological activities of apilarnil are attributed to its complex chemical profile, which includes fatty acids, flavonoids, glycerophospholipids, alcohols, sugars, amino acids, and steroid compounds [16,17]. Aida et al. investigated the chemical composition and nutritional potential of apilarnil and identified 44 compounds, of which 30 volatile compounds were classified as esters, ketones, ethers, alcohols, fatty acids, aldehydes, amines, and alkenes. They also reported that oleic acid (64.75%) and palmitic acid (26.08%) were the predominant lipid components [17]. In another study, İnci et al. analyzed the phytochemical content and biological activities of apilarnil and reported that quinic acid, fumaric acid, kaempferol, and quercetin were the most abundant compounds when expressed as mg analyte per gram of dry extract [16].

This study has several limitations. First, as an experimental animal study, the findings may not be directly generalizable to human fracture healing. Second, serum biomarkers related to bone metabolism and detailed molecular analyses were not performed. Third, the relatively small sample size and short follow-up period may have limited the statistical power of the study, particularly for non-significant outcomes. Biomechanical testing was not performed on postoperative day 15 due to insufficient hard callus formation, resulting in the absence of early mechanical data. Finally, the absence of a positive control group and the lack of detailed phytochemical characterization of apilarnil indicate that this study should be considered an exploratory investigation. Furthermore, only a single dose of apilarnil was evaluated; therefore, dose–response relationships and the optimal therapeutic dose could not be determined. Further studies with larger sample sizes, multiple dose regimens, and more comprehensive molecular analyses are needed to clarify these findings.

## 5. Conclusions

Apilarnil demonstrated supportive effects on fracture healing by improving early histopathological repair and enhancing biomechanical strength during the later stages of bone regeneration in a rat femoral fracture model. Although no significant radiological differences were observed, the findings suggest that apilarnil may contribute to the biological and mechanical aspects of fracture repair. As a bioactive bee-derived natural product, apilarnil may represent a potential supportive strategy for bone healing. However, further experimental and clinical studies are required to clarify its mechanisms of action and potential therapeutic relevance.

## Figures and Tables

**Table 1:** Histopathological comparison of control and apilarnil-treated groups on postoperative days 15, 30 and 45.

Histopathological Evaluation		Control	Apilarnil	*p* *
**Day 15**	Mean ± SD	2.14 ± 1.07	3.71 ± 1.80	**0.042**
	Median (IQR)	3 (1–3)	4 (3–4)	
**Day 30**	Mean ± SD	7.00 ± 1.63	8.43 ± 2.15	0.079
	Median (IQR)	7 (6–8)	9 (8–10)	
**Day 45**	Mean ± SD	8.14 ± 2.91	9.00 ± 1.53	0.631
	Median (IQR)	9 (7–10)	10 (8–10)	
	***p*** **	**0.004**	**0.003**	
Dunn’s Multiple Comparison test		Control	Apilarnil	
**Day 15/Day 30**		**0.017**	**0.027**	
**Day 15/Day 45**		**0.018**	**0.018**	
**Day 30/Day 45**		0.308	0.655	

* Mann Whitney U test, ** Friedman test, IQR: interquartile range, ±SD: standard deviation.

**Table 2:** Biomechanical comparison of control and apilarnil-treated groups on postoperative days 30 and 45.

Biomechanics		Control	Apilarnil	*p* *
**Day 30**	Mean ± SD	45.0 ± 14.7	151.4 ± 64.4	**0.001**
	Median (IQR)	40 (35–50)	160 (80–200)	
**Day 45**	Mean ± SD	85.7 ± 17.2	172.9 ± 50.9	**0.004**
	Median (IQR)	80 (70–90)	180 (110–200)	
	***p*** **	**0.028**	0.292	

* Mann Whitney U test, ** Wilcoxon test, IQR: interquartile range, ±SD: standard deviation.

**Table 3:** Radiological comparison of control and apilarnil-treated groups on postoperative days 15, 30 and 45.

Radiological Evaluation		Control	Apilarnil	*p* *
**Day 15**	Mean ± SD	1.43 ± 0.54	1.29 ± 0.49	0.591
	Median (IQR)	1 (1–2)	1 (1–2)	
**Day 30**	Mean ± SD	3.29 ± 0.95	3.14 ± 1.07	0.782
	Median (IQR)	4 (2–4)	3 (3–4)	
**Day 45**	Mean ± SD	3.71 ± 0.49	3.71 ± 0.76	0.656
	Median (IQR)	4 (3–4)	4 (4–4)	
	***p*** **	**0.004**	**0.002**	
Dunn’s Multiple Comparison test		Control	Apilarnil	
**Day 15/Day 30**		**0.026**	**0.023**	
**Day 15/Day 45**		**0.016**	**0.017**	
**Day 30/Day 45**		0.276	**0.046**	

* Mann Whitney U test, ** Friedman test, IQR: interquartile range, ±SD: standard deviation.

## Data Availability

The datasets used and/or analysed during the current study are available from the corresponding author (Raşit Emin Dalaslan) on reasonable request.

## Abbreviation

The following abbreviations are used in this manuscript:

DLH	Drone Larvae Homogenate
